# Effects of different information brochures on women’s decision-making regarding mammography screening: study protocol for a randomized controlled questionnaire study

**DOI:** 10.1186/1745-6215-14-319

**Published:** 2013-10-01

**Authors:** Elisabeth Gummersbach, Jürgen in der Schmitten, Heinz-Harald Abholz, Karl Wegscheider, Michael Pentzek

**Affiliations:** 1 University of Duesseldorf, Medical Faculty, Institute of General Practice, Moorenstrasse 5, Dusseldorf D-40225, Germany; 2 Medical Faculty, Department of Medical Biometry and Epidemiology, University of Hamburg, Hamburg D-20251, Germany

**Keywords:** Information brochure, Mammography screening, Informed consent, Decision making

## Abstract

**Background:**

In order to give informed consent for mammography screening, women need to be told the relevant facts; however, screening information often remains vague because of the worry that detailed information might deter women from participating in recommended screening programs. Since September 2010, German women aged 50 to 69 invited for mammography screening have received a new, comprehensive information brochure that frankly discusses the potential benefit and harm of mammography screening. In contrast, the brochure that was in use before September 2010 contained little relevant information.

The aim of this study is to compare the impact of the two different brochures on the intention of women to undergo mammography screening, and to broaden our understanding of the effect that factual information has on the women’s decision-making.

**Methods:**

This is a controlled questionnaire study comparing knowledge, views and hypothetical preferences of women aged 48–49 years after receiving the old versus the new information brochure. German GP’s in the region of North Rhine-Westfalia will be asked by mail and telephone to participate in the study. Eligible women will be recruited via their general practitioners (GPs) and randomized to groups A ('new brochure’) and B ('old brochure’), with an intended recruitment of 173 participants per group. The study is powered to detect a 15% higher or lower intention to undergo mammography screening in women informed by the new brochure.

**Discussion:**

This study will contribute to our understanding of the decision-making of women invited to mammography screening. From both ethical and public health perspectives, it is important to know whether frank, factual information leads to a change in the intention of women to participate in a recommended breast cancer screening program.

**Trial registration:**

DRKS00004271

## Background

Since 2005, all women in Germany between 50 and 69 years of age have been invited to the national mammography screening program. From the public health perspective, there is a legitimate interest in achieving a high participation rate. Some are worried that the number of participants might decrease considerably if fact- and figure-based information is given to screening participants, because the benefit for the individual participant is very small.

On the other hand, it is an ethical duty to inform people truthfully and intelligibly about the screening in order to allow them to make informed decisions. The patients’ right to disclosure of all relevant information for medical decisions is fixed in the European Charter of Patients’ Rights [1]. The operationalization of this right builds the theoretical background of this study: For an informed choice (evidence-based patient choice) patients need evidence-based patient information (EBPI). This information has to include risks, benefits, number needed to screen, sensitivity, specificity and so on [2]. Further theoretical input comes from O’Connor’s concept of decisional conflict describing the weighing of risks and benefits in case of uncertainty about outcomes [3].

Information brochures are one way of giving information to patients. The brochure distributed to eligible women until September 2010, together with the invitation letter for mammography screening ('old brochure’), contained hardly any material relevant to making an informed decision [4,5]. Following increasing criticism, the 'old brochure’ was replaced by a thoroughly revised version ('new brochure’) which contains considerably more information about the benefits and risks of the screening and also illustrates the facts with numerical examples [4]. From an informed-consent perspective, this new brochure can be judged exemplary in patient information for screening examinations. To our knowledge, neither in Germany nor in other countries is there any official information material on screening examinations that informs as comprehensively as this one [6-9].

Against this background it is very important to know whether more detailed and comprehensive information will effect differences in the intention of women to participate in breast cancer screening. Moreover, we know little about the role that information brochures play overall in the decision to participate or not to participate in screening programs.

### Research questions

We wanted to know whether, and to what extent, the content of the information brochure distributed nationwide in Germany influences women’s intention to participate in mammography screening, and whether EBPI-relevant, transparent information regarding the potential benefits and harms of early detection screening influences this intention. In detail, our research questions are:

1. What influence does an information brochure including relevant facts and figures on the harms and benefits of mammography screening ('new brochure’) have on women’s intention to take part in screening, compared to a brochure that mainly promotes the screening while omitting relevant information ('old brochure’)?

2. Do different information brochures lead to different knowledge about the potential benefits and harms of mammography screening?

3. Are there any other factors (beside the contents of brochures) influencing the decision to take part (for example, personal experience with breast cancer, marital status)?

### Hypothesis

The two groups ('new’ versus 'old brochure’) differ in their intention to take part in mammography screening.

### Current status of research

There are only few studies on the topic, and as it is ethically problematic to perform a study within the context of a real invitation for screening, the question whether the tested persons would participate if they were invited has been asked only hypothetically in those studies.

There are several studies examining decision-making for mammography screening programs. Some of them investigate optimized decision aids, others - as we do - look at information brochures that are in use by national screening programs [10-15]. Webster *et al.* performed a questionnaire study in England in 2006. They interviewed 1,000 women aged 48 to 64 years about their knowledge and their attitude toward mammography screening (baseline assessment) [15]. They repeated the questionnaire study in 2007 with 100 women who were recruited from the baseline group, this time after having read the recently implemented information brochure about mammography screening. The second assessment showed a significant gain in knowledge [6]. Mathieu *et al.* investigated the effect of 'online decision aids’ for mammography screening on 321 Australian women aged 70, and subsequently on 38 to 40 years old women. In both studies, the women in the intervention group - those with the decision aid - had more knowledge about the screening and showed greater decision competence than the group without such an aid, and they refused to participate in screening more often [14,16]. A non-comparative descriptive study was performed by Börgermann using an information brochure for PSA screening: 1,537 men within a preventive check-up had been interviewed using a questionnaire. Although 82% of the men felt well informed after they had read the brochure, their knowledge tested in the questionnaire was rather poor [17]. In a study by Steckelberg *et al.* on decision-making for colonoscopy screening, a specially produced brochure for such screening containing pertinent evidence-based information was compared with a standard information brochure with regard to willingness, knowledge and attitudes of the interviewees. The study was performed on the target group (age 50 to 75 yrs). The results showed that the intervention group had better knowledge; although a 'positive attitude’ towards the screening was significantly less common in this group, no difference in willingness to participate could be demonstrated [8].

## Methods

This is a controlled two-armed single-blind questionnaire study. Women will be randomized into two groups: group A will receive the 'new brochure’ (Additional file 1) with facts and figures corresponding to current evidence on outcome. Group B will receive the 'old brochure’ (Additional file 2) with little, merely promoting, information.

We are addressing women aged 48 to 49 yrs, that is, one or two years before eligibility for mammography screening. For ethical reasons we did not choose the eligible age, so as not to influence the immediate decision of the women regarding participation in screening. Also, women aged 50+ are more likely to have received and studied the 'new brochure’ already, so the study’s control arm receiving the 'old brochure’ would probably have been contaminated. The women are recruited via their GPs, whom we contact with a request to participate in the study. Twenty-five of 106 doctors contacted in the North Rhine-Westphalia area of Germany agreed to participate in the study and to send us a list of all women of eligible age who had presented to their practice in the past two years. In Germany, women of this age group (48 to 49 years) contact their GP about four times a year on average [18].

To pseudonymize the women, all names are deleted from the list before it is sent to us, while the internal practice reference number remains for later identification. We then randomly select 24 women from each practice for inclusion in the study. Randomization is at patient level, that is, these 24 women are randomized per practice into two groups; A ('new brochure’) or B ('old brochure’). According to the random allocation, the women in groups A and B will receive the respective brochure, a letter, and a uniform questionnaire about knowledge and intention to take part in a screening. Closed envelopes which are marked only by the internal practice reference number are sent to the practices and from there directly to the women. With the attached letter they are asked to complete the questionnaire after reading the brochure and to send it back to us, without disclosing their identity, for pseudonymous analysis. The questionnaires contain a reference number that can only be decoded by the GPs.

### Instruments

#### ***Information brochures***

The women receive either the 'old’ or the 'new’ information brochure attached to the invitation for mammography screening prior to or after the year 2010, respectively. The 'old brochure’ promotes the screening and gives only limited information about facts and figures, whereas the 'new brochure’ provides transparent information about the potential benefits and harms of the screening based on the available evidence, illustrated by figures (Table 1).

**Table 1:** Criteria for an informed screening choice and their consideration in brochure A ('new’) and B ('old’)

**Criteria**	**Brochure A**	**Brochure B**
1. Rate of pathological result of screening	Y	Y^a^
2. Benefit of mammography screening	Y	Y
3. Reduction of mortality by screening	Y^a^	N
4. Absolute/relative risk reduction	N	N
5. Reduction of total mortality	N	N
6. Sensitivity of screening	Y	Y
7. Specificity of screening	Y	N
8. How many women have to be screened to save one from dying of breast cancer (NNS^b^)	Y^a^	N
9. Recommendation to self-checking the breast (BSE^c^)	N^d^	Y^d^
10. Overdiagnosis - early cancer (How many DCIS^e^ are discovered that would never have any clinical relevance?)	Y^a^	N
11. Rate of false positive results	Y^a^	N
12. Increase of operation and radiation of women who do not benefit	Y	N
13. Interval cancer	Y	Y
14. Earlier diagnosis without delay of death	Y	N
15. Potential side effects of x-ray	N	Y

^a^Includes specification of figures.

^b^NNS: number needed to screen.

^c^BSE: breast self examination.

^d^Studies have shown that BSE has no effect on the mortality of breast cancer, therefore a positive recommendation must be judged negatively with view to an informed choice.

^e^DCIS: ductal carcinoma *in situ*.

Formal description of the two brochures:

The design of information brochures is a scientific issue of its own [19]; we did not have any influence on the design of the two brochures. Both brochures appear in the same standard, on edge flyer format. They both comprise 12 pages, and none of them contains any figures or pictograms. A comparing analysis of formal criteria of the two brochures has not been provided so far. In our study we did not examine the effects of the formal and graphical design of the brochures [2].

#### ***Questionnaire***

The questionnaire employed to elicit the women’s knowledge has been developed on the basis of already existing, partly validated questionnaires [12-15,17] and builds upon the criteria for relevant knowledge to make an informed decision [2] (Additional file 3). It contains five questions on *knowledge* (one point for every correct answer) [2,14]. Two questions measure *the women’s self-assessment of their knowledge* about mammography screening; two questions are about the perceived *relevance of knowledge* for the individual [12]. There is one yes/no question on the intention to participate (primary outcome), combined with a confidence rating for this decision (six-point scale from 'very unsure’ to 'absolutely sure’). Furthermore, there is one question each about personal experience with breast cancer, educational level, age, marital status and native language, two questions concerning the assessment of the brochure, and an open question for the participant to indicate information needs not sufficiently dealt with in the respective brochure. For secondary analyses we constructed items on the following topics: preferred media for support in screening decision, risk perception, attitudes towards mammography screening.

The questionnaire was pre-tested in 15 women with regard to comprehensibility, resulting in slight revisions of item wording and scaling.

### Inclusion and exclusion criteria

All women aged 48 or 49 years identified by the participating practices will be included. This age group has not yet been invited to a mammography screening and has not so far made the decision to participate in such screening. However, the first invitation is imminent, so the subject matter is already of interest for them. GPs are asked to exclude women from their lists who are not sufficiently fluent in German, and women with obvious cognitive limitations. Compliance with the exclusion criteria remains in the responsibility of the inviting physicians.

### Consent and ethical approval

All participants will be asked for informed consent to participate.

The study was approved by the ethics committee of the University Hospital, Heinrich-Heine-University of Duesseldorf (22.02.2012, ref. number 3797) (Additional file 4).

### Sample size

At present, the participation rate for mammography screening in North Rhine-Westphalia, Germany is 53.7% [20]. We assume that participation in this interview study is more likely than participation in real screening, and therefore expect a 60% willingness to take part on mammography screening in the interviews. We judge a group difference of at least 15 absolute percentage points (that is, from 60% to 75% or from 60% to 45%) to be relevant. To detect such differences in the primary analysis with a power of 80%, 173 study participants per group = 346 participants have to be included. We expect a response rate of approximately 50%, so at least 692 women need to be contacted.

### Statistical analysis

The primary analysis will include all women who consented to study participation and gave information on her intention to participate in a potential mammography screening (full analysis set). It consists of a likelihood ratio chi-square test of the intention to screening participation with a two-sided alpha of 5%. As a sensitivity analysis, best and worst case analyses will be performed in all consenting women (intention-to-treat population) with different imputations for missing values. In a second step, a logistic regression model including potential confounders and effect modifiers (for example, knowledge) will be fitted to the data for further explanation of the primary result. Secondary endpoints (knowledge, self-assessed knowledge, importance of knowledge, confidence in decision) will be compared using chi-square tests or Mann–Whitney *U* tests, whichever appropriate.

### Limitations

A possible influence of differences in the formal design of the two brochures on the decision to take part in the screening program is not subject of our examination; however, the two brochures are formally quite similar.

## Trial status

We are currently recruiting participants.

## Abbreviations

GP: General practitioner; EBPI: Evidence based medicine; NNS: Number needed to screen; BSE: Breast self examination; DICS: Ductal carcinoma *in situ*.

## Competing interests

There are no competing interests.

## Authors’ contributions

EG conceived the study, participated in the sequence alignment and wrote the manuscript. She is responsible for recruiting participants. MP revised the manuscript and participated in the sequence alignment and developing the questionnaire. HHA contributed to the conception of the study, participated in the design of the study and in the sequence alignment. JidS participated in the development of the questionnaire and revised the manuscript. KW performed the statistical analysis. All authors read and approved the final manuscript.

## Supplementary Material

Additional file 1 neu mammo_merkblatt.pdf (new brochure). Click here for file

Additional file 2 alt mammo_merkblatt.pdf (old brochure). Click here for file

Additional file 3 Fragebogen 2 englisch.doc (questionnaire). Click here for file

Additional file 4 Statement Ethikkommission.pdf (statement ethics committee of the university hospital, Heinrich-Heine University of Duesseldorf). Click here for file
