# Supplementary material for: Effects of different information brochures on women’s decision-making regarding mammography screening: study protocol for a randomized controlled questionnaire study
Source: Trials. 2013 Oct 1;14:319. doi: 10.1186/1745-6215-14-319 (PMC3851440; doi:10.1186/1745-6215-14-319)
Supplement: Additional file 1 — neu mammo_merkblatt.pdf (new brochure). [file 1745-6215-14-319-S1.pdf]

HEINRICH HEINE
UNIVERSITÄT DÜSSELDORF

ETHIKKOMMISSION der Medizinischen Fakultät

ETHIKKOMMISSION der Medizinischen Fakultät der Heinrich-Heine-Universität, Moorenstr. 5, 40225 Düsseldorf

Frau
Dr. med. E. Gummersbach
Institut für Allgemeinmedizin
Geb. 14.97

HIER

Prof. Dr. T. Hohlfeld

Telefon: (0211)81-19590
Sekretariat: (0211)81-19591
Fax: (0211)81-19592
Ethikkommission@med.uni-duesseldorf.de

22. Feb. 2012

Stets angeben:
Studiennummer: 3797

Sehr geehrte Frau Kollegin Gummersbach,

die Ethikkommission der Medizinischen Fakultät der Heinrich-Heine-Universität Düsseldorf hat das von Ihnen vorgelegte Studienprotokoll mit dem Titel:

Welchen Einfluss haben Informationsbroschüren auf die Entscheidung der Frau am Mammographie-Screening teilzunehmen

geprüft und beurteilt.

Von Seiten der Kommission bestehen keine ethischen oder rechtlichen Bedenken gegen die Durchführung der geplanten Studie.

Nach Abschluss des Projektes bitte ich um Übersendung eines knappen Schlussberichtes oder einer abschließenden Publikation.

Für die Durchführung der Studie wünschen wir viel Erfolg!

Mit freundlichen Grüßen

Prof. Dr. Klaus-Dietrich Kröncke
i. A. der Kommission

Rechtsmittelbelehrung: Gegen diesen Bescheid kann innerhalb eines Monats nach Bekanntgabe Widerspruch erhoben werden. Der Widerspruch ist beim Dekan der Medizinischen Fakultät der Heinrich-Heine-Universität Düsseldorf schriftlich einzulegen.

HEINRICH HEINE
UNIVERSITÄT DÜSSELDORF

ETHIKKOMMISSION der Medizinischen Fakultät

ETHIKKOMMISSION der Medizinischen Fakultät der Heinrich-Heine-Universität, Moorenstr. 5, 40225 Düsseldorf

Frau
Dr. med. E. Gummersbach
Institut für Allgemeinmedizin
Geb. 14.97

HIER

Prof. Dr. T. Hohlfeld

Telefon: (0211)81-19590
Sekretariat: (0211)81-19591
Fax: (0211)81-19592
Ethikkommission@med.uni-duesseldorf.de

22. Feb. 2012

Stets angeben:
Studiennummer: 3797

Sehr geehrte Frau Kollegin Gummersbach,

die Ethikkommission der Medizinischen Fakultät der Heinrich-Heine-Universität Düsseldorf hat das von Ihnen vorgelegte Studienprotokoll mit dem Titel:

Welchen Einfluss haben Informationsbroschüren auf die Entscheidung der Frau am Mammographie-Screening teilzunehmen

geprüft und beurteilt.

Von Seiten der Kommission bestehen keine ethischen oder rechtlichen Bedenken gegen die Durchführung der geplanten Studie.

Nach Abschluss des Projektes bitte ich um Übersendung eines knappen Schlussberichtes oder einer abschließenden Publikation.

Für die Durchführung der Studie wünschen wir viel Erfolg!

Mit freundlichen Grüßen

Prof. Dr. Klaus-Dietrich Kröncke
i. A. der Kommission

Rechtsmittelbelehrung: Gegen diesen Bescheid kann innerhalb eines Monats nach Bekanntgabe Widerspruch erhoben werden. Der Widerspruch ist beim Dekan der Medizinischen Fakultät der Heinrich-Heine-Universität Düsseldorf schriftlich einzulegen.
