# Supplementary material for: Effects of different information brochures on women’s decision-making regarding mammography screening: study protocol for a randomized controlled questionnaire study
Source: Trials. 2013 Oct 1;14:319. doi: 10.1186/1745-6215-14-319 (PMC3851440; doi:10.1186/1745-6215-14-319)
Supplement: Additional file 2 — alt mammo_merkblatt.pdf (old brochure). [file 1745-6215-14-319-S2.doc]

The following is a translation of the German questionnaire employed in the mammography study by E. Gummersbach et al. (2012/13), Institute of General Practice, University of Düsseldorf.

Please note: This translation has not been revised by a native speaker, let alone a native speaker expert in questionnaire methodology; it serves the purpose of scientific transparency and communication of our approach, and should not be used as a survey instrument, or semantically scrutinized.

**QUESTIONNAIRE**

**on mammography screening**

**Dear participant**!

You have been asked to read an information brochure on mammography screening.

Now we turn to you with the request to reply to a number of questions with regard to mammography screening. This questionnaire is anonymous so please mind not to put your name on it.

**What am I to do?**

Please note that not all questions relate directly to the brochure, but rather to your general views on mammography screening. So please don’t be surprised if you encounter some questions that seem not to have been subject of the brochure.

**Do not worry about „right“ or „wrong“;
this is not a test, and you are not being examined!**

**Please do always tick the answer that seems to best fit for you personally!**

**And please carefully read the entire question before you start answering it.**

**Let’s go!**

***First of all, we have two general questions with regard to mammography screening***

***Please tick only ONE answer per question!***

1. **My understanding of the risks and potential side effects of mammography screening is…**

| … very poor | … fairly poor | … fairly good | … very good |
| --- | --- | --- | --- |
| ❑ | ❑ | ❑ | ❑ |

1. **My understanding of the advantages of the mammography screening is…**

| … very poor | … fairly poor | … fairly good | … very good |
| --- | --- | --- | --- |
| ❑ | ❑ | ❑ | ❑ |

*→ weiter*

***How relevant are the risk and potential side effects of the mammography screening for you?***

***Again, please tick again only the ONE answer that seems to fit best to you.***

1. **For me, knowing the risks of the mammography screening is …**

| … totally **un**important | … fairly **un**important | … fairly important | … absolutely important |
| --- | --- | --- | --- |
| ❑ | ❑ | ❑ | ❑ |

***How relevant are the advantages of the screening for you?***

***Again, please tick again only the ONE answer that seems to fit best to you.***

1. **For me, knowing the advantages of the mammography screening is …**

| … totally **un**important | … fairly **un**important | … fairly important | … absolutely important |
| --- | --- | --- | --- |
| ❑ | ❑ | ❑ | ❑ |

***In Germany, women aged 50-65 years are invited to participate in the mammography screening every two years. Please estimate the benefit and potential side-effects.***

1. **Imagine 100 women who have participated in a mammography screening.
   Now what do you think: How many of these 100 women will need to undergo a further diagnostic procedure because the mammography was obtrusive, i.e. suspicious of breast cancer?**

***Please tick only ONE answer!***

| **About 1** of the 100 women | **About 10** of the 100 women | **About 20** of the 100 women | **About 50** of the 100 women |
| --- | --- | --- | --- |
| ❑ | ❑ | ❑ | ❑ |

***In mammography screening, there are findings that are suspicious of breast carcinoma so that it needs to be clarified by means of further diagnostic procedures whether the tissue change is truly cancer or in fact harmless.***

1. **Imagine that 10 women who underwent mammography screening are being told that there is a suspicious finding, and asked to undergo another diagnostic procedure. Now what do you think, how many of these 10 women are afterwards actually diagnosed to have breast cancer?  *Please tick only ONE answer!***

| **1** of 10 women | **3** of 10 women | **7** of 10 women | **all 10** women |
| --- | --- | --- | --- |
| ❑ | ❑ | ❑ | ❑ |

*→ weiter*

***Please note: The following questions 7 to 9 relate to a time span of 10 years.***

1. **Now please imagine 1,000 women aged 50 years.**

**What do you think: Approximately how many of these 1,000 women aged 50 will catch breast cancer within the next ten years?
*Please tick only ONE answer!***

| **20** of the 1,000 women | **50** of the 1,000 women | **100** of the 1,000 women | **300** of the 1,000 women |
| --- | --- | --- | --- |
| ❑ | ❑ | ❑ | ❑ |

1. **Now please imagine another 1,000 women who over a time period of 10 years have undergone mammography every 2 years.**

**What do you think: Approximately how many of these 1,000 women were saved from dying of breast cancer thanks to the regular screening?
*Please tick only ONE answer!***

| approx. **2** of these 1,000 women | approx. **15** of these 1,000 women | approx. **100** of these 1,000 women | approx. **200** of these 1,000 women |
| --- | --- | --- | --- |
| ❑ | ❑ | ❑ | ❑ |

1. **Of 1,000 women who between the age of 50 and 60 years participated in mammography screening every 2 years, 24 are diagnosed with breast cancer at some point in time during this decade.**

**In some of these women, the cancer will be detected by means of the screening; however, in other women it will be detected between 2 screenings.**

**What do you think: In how many of these 24 women the breast cancer will be detected not by means of the screening but between 2 screenings?**

***Please view the below figures and tick what you think is most likely to be correct!***

***Please tick only ONE answer!***

| ❑ | ❑ | ❑ | ❑ |
| --- | --- | --- | --- |

in 1 of 24

in 3 of 24

in 6 of 24

in 9 of 24

= Breast cancer detected NOT by the screening,

but in the time between 2 screenings.

= Breast cancer detected by mammography screening

1. **Compared with most other women of your age: How high do you estimate your personal lifetime risk to develop breast cancer?**

***Please tick only ONE answer!***

- I estimate my personal lifetime risk for breast cancer to be **lower** than average.
- I estimate my personal lifetime risk for breast cancer to be around **average**.
- I estimate my personal lifetime risk for breast cancer to be **higher** than average.

Please explain the reasons of your estimation: ....................................................................

*→ continue overleaf!*

1. **What is your opinion?
   Please appraise the following statements on a scale of 1 (not at all sensible) to 6 (very sensible)**

***Please tick only ONE answer!***

**a. In principle, my opinion is that participation in mammography screening is …**

| *…not at all sensible* | 1  ❑ | 2  ❑ | 3  ❑ | 4  ❑ | 5  ❑ | 6  ❑ | *… very sensible* |
| --- | --- | --- | --- | --- | --- | --- | --- |

**b. For me personally, participation in mammography screening would be**

| *…not at all important* | 1  ❑ | 2  ❑ | 3  ❑ | 4  ❑ | 5  ❑ | 6  ❑ | *…very important* |
| --- | --- | --- | --- | --- | --- | --- | --- |

| *…not at all* ***un****pleasant* | 1  ❑ | 2  ❑ | 3  ❑ | 4  ❑ | 5  ❑ | 6  ❑ | *…very* ***un****pleasant* |
| --- | --- | --- | --- | --- | --- | --- | --- |

1. **Please make a decision: If you were invited to the mammography screening today, would you participate?  *Please tick only ONE answer!***

**❑ No**

**❑ Yes**

**How confident are with this decision?**

| *very* ***un****confident* | 1  ❑ | 2  ❑ | 3  ❑ | 4  ❑ | 5  ❑ | 6  ❑ | *very  confident* |
| --- | --- | --- | --- | --- | --- | --- | --- |

1. **What do you think – what would be the main factors that might influence your decision for or against undergoing mammography screening?
   *Please tick only ONE answer!***

- My physician’s recommendation
- Reports in newspapers, TV or internet
- Informations provided in the invitation brochure
- Friends and family
- Personal experiences with women who suffer(ed) from breast cancer
- Other (please specify):

………………………………………………………………………………….

*→ weiter*

***Remember, we have sent you an information brochure on mammography screening. Please let us know your opinion:***

1. **How do you judge the amount of information provided by the brochure?
   *Please tick only ONE answer!***

**For me, the brochure provided …**

- …rather not enough information
- …the right amount of information
- …rather too much information (if anything I feel confused by the brochure)

1. **Did the brochure help you to decide whether you want to participate in mammography screening in the near future or not?**

***Please tick only ONE answer!***

**For my decision making, the brochure was…**

| …of no help at all | …of little help | ... of some help | …very helpful |
| --- | --- | --- | --- |
| ❑ | ❑ | ❑ | ❑ |

1. **Please rate the brochure by means of a global judgment: How do you find it?**

| ❑ | ❑ | ❑ | ❑ | ❑ | ❑ |
| --- | --- | --- | --- | --- | --- |
| **1** | **2** | **3** | **4** | **5** | **6** |
| **very good** | **good** | **satisfactory** | **sufficient** | **poor** | **fail** |

If you wish, please share with us the reason(s) for your rating:

_____________________________________________________________________

_____________________________________________________________________

***Finally, we have some questions with regard to your person:***

1. **Did you ever make personal experiences with breast cancer?**

***Please tick only ONE answer!***

- No, I do not know anybody personally who suffers / suffered from breast cancer
- Yes, a friend or distant relative has / had breast cancer
- Yes, one of my close relatives (grandmother, mother, sister, daughter) has / had breast cancer.
- Yes, I am / have been suffering from breast cancer myself

*→ weiter*

1. **What is your age?**

**………… years**

1. **What is your mother tongue?**

- German
- Turkey
- Russian
- Polish
- Other, please specify: …………………………………..

1. **What is your family status?**

- unmarried
- married
- divorced
- widowed

1. **Do you live alone, or together with other persons in a common household?**

***More than one answer possible***

- alone
- with partner / spouse
- with (a) relative(s)
- with others

1. **What is your highest educational qualification?**

- no graduation from school
- certificate of primary education
- certificate of secondary education
- A levels
- graduation from university
- other graduation (please specify):___________________________

1. **Now our last question, coming back to mammography screening:
   Would you wish the brochure to contain more information with regard to certain aspects of the mammography screening?**

- no
- yes

If yes, please specify:

.........................................................................................................................................
