# Supplementary material for: Effects of different information brochures on women’s decision-making regarding mammography screening: study protocol for a randomized controlled questionnaire study
Source: Trials. 2013 Oct 1;14:319. doi: 10.1186/1745-6215-14-319 (PMC3851440; doi:10.1186/1745-6215-14-319)
Supplement: Additional file 3 — Fragebogen 2 englisch.doc (questionnaire). [file 1745-6215-14-319-S3.pdf]

Gemeinsamer
Bundesausschuss

Weitere Informationen erhalten Sie ebenfalls unter www.mammo-programm.de oder bei der einladenden Stelle (Zentrale Stelle). Fragen zur Einladung beantwortet Ihnen Ihre Zentrale Stelle.

Der Gemeinsame Bundesausschuss (G-BA) nach § 91 Abs. 5 SGB V beschließt die Krebsfrüherkennungs-Richtlinie. Diese ist auf der Website des Gemeinsamen Bundesausschusses einsehbar.

Das Merkblatt ist Bestandteil der Krebsfrüherkennungs-Richtlinie (Beschluss vom 15.12.2003) und klärt über Hintergründe, Ziele, Inhalte, Vorgehensweise und Datenschutz des Programms zur Früherkennung von Brustkrebs auf.

www.mammo-programm.de
www.g-ba.de

Herausgeber: Gemeinsamer Bundesausschuss, Stabsbereich Öffentlichkeitsarbeit und Kommunikation | Layout: Braun Grafikdesign, Berlin | Juni 2010

Informationen zum MAMMOGRAPHIE-SCREENING

Programm zur Früherkennung von Brustkrebs für Frauen zwischen 50 und 69 Jahren

MAMMOGRAPHIE SCREENING PROGRAMM

Gemeinsamer
Bundesausschuss

WORUM GEHT ES?

Mit der Einladung zum Mammographie-Screening erhalten Sie dieses Merkblatt. Es dient dazu, Sie über Brustkrebs allgemein sowie über die Möglichkeiten zur Früherkennung von Brustkrebs zu informieren. Sie sollen sich mit Hilfe des Merkblatts selbst eine Meinung bilden, ob Sie die Einladung annehmen möchten. Die Teilnahme am Programm ist freiwillig.

Wenn Sie zwischen 50 und 69 Jahre alt sind, haben Sie alle zwei Jahre Anspruch auf eine Mammographie-Untersuchung zur Früherkennung von Brustkrebs. Die Kosten werden von der gesetzlichen Krankenversicherung übernommen und Sie müssen keine Praxisgebühr bezahlen. Sind Sie privat versichert, fragen Sie bitte Ihre Krankenversicherung zur Kostenübernahme.

Screening bedeutet, dass allen Menschen einer Altersgruppe eine bestimmte Untersuchung angeboten wird. Bei dem Mammographie-Screening wird die weibliche Brust geröntgt. Ziel der Untersuchung ist es, Brustkrebs möglichst früh zu entdecken, um ihn noch erfolgreich behandeln zu können. Eine Entstehung von Brustkrebs kann dadurch jedoch nicht verhindert werden.

WAS ZEICHNET DAS PROGRAMM AUS?

In Deutschland wurde mit großem Aufwand ein Mammographie-Screening-Programm eingerichtet. Das Mammographie-Programm ist ein zusätzliches Angebot zu der jährlichen Krebsvorsorgeuntersuchung bei der Frauenärztin oder dem Frauenarzt.

Das Mammographie-Screening-Programm in Deutschland erfüllt die strengen Qualitätskriterien der „Europäischen Leitlinien“:

- → Die Mammographie wird von Fachkräften an streng kontrollierten, modernen Geräten durchgeführt.
- → Jede Mammographie-Aufnahme wird von mindestens zwei Ärztinnen oder Ärzten begutachtet, die Mammographien von mindestens 5.000 Frauen pro Jahr beurteilen.
- → Auffällige Befunde werden innerhalb des Früherkennungs-Programms von speziell fortgebildeten Ärztinnen und Ärzten abgeklärt.

WAS IST BRUSTKREBS?

Wenn sich eine Zelle des Brustgewebes unkontrolliert zu teilen beginnt, kann Krebs heranwachsen, in gesundes Gewebe eindringen und Absiedelungen, so genannte Metastasen, bilden. Jährlich erkranken in Deutschland etwa 57.000 Frauen an Brustkrebs. Im Alter zwischen 50 und 69 Jahren ist es etwa eine von 20 Frauen. Das durchschnittliche Erkrankungsalter liegt bei 63 Jahren. Rund 17.500 Frauen sterben jährlich an Brustkrebs, im Alter zwischen 50 und 69 Jahren etwa eine von 80 Frauen.

Brustkrebs ist so vielfältig wie kaum eine andere Krebsart. Manche Brustkrebstypen entwickeln sich langsam und neigen kaum dazu, Metastasen zu bilden, andere sind dagegen sehr aggressiv.

Ein häufiger Tumortyp ist das so genannte Duktale Karzinoma in Situ (DCIS), das in der Mammographie besonders gut entdeckt wird, das sich jedoch nur in etwa einem von drei Fällen zu einem gefährlichen Tumor weiter entwickelt. Da sich nicht vorhersagen lässt, welcher sich weiter entwickelt, werden alle DCIS behandelt.

WELCHE RISIKOFAKTOREN GIBT ES?

Mit dem Alter steigt das Risiko an Brustkrebs zu erkranken. Ist Ihre Mutter, Tochter oder Schwester von Brustkrebs betroffen – verdoppelt sich Ihr Risiko. Sind zwei Verwandte betroffen, vervierfacht sich das Risiko. Für folgende weitere Faktoren ist gesichert, dass sie das Brustkrebsrisiko erhöhen oder senken: Zu einem etwas höheren Risiko, an Brustkrebs zu erkranken, führen starker Alkoholkonsum, Röntgenstrahlen, Medikamente zur Hormontherapie in den Wechseljahren sowie Übergewicht nach den Wechseljahren. Stillen dagegen vermindert das Risiko. Keine Rolle spielen psychische Faktoren, wie die innere Einstellung, Lebensfreude oder Stress.

WIE LÄUFT DAS SCREENING AB?

Den Brief, der Sie zur Screening-Untersuchung einlädt, versendet die so genannte „Zentrale Stelle“. Diese erhält Ihr Geburtsdatum und Ihre Anschrift vom örtlichen Einwohnermelderegister.

Die Untersuchung findet an einem Standtort der Screeningeinheit in Ihrer Region statt, manchmal auch in speziell dafür eingerichteten Fahrzeugen. Eine Screening-Einheit wird von besonders fortgebildeten und erfahrenen Ärztinnen und Ärzten geleitet.

Eine Mammographieaufnahme zu machen gehört – wie jede andere Röntgenuntersuchung – zu den Aufgaben von medizinischen Röntgen-Fachkräften. Um im Screening-Programm arbeiten zu können, müssen sie sich besonders qualifizieren. Falls Sie vor Ort eine medizinische Frage haben, die Ihre Röntgenassistentin nicht beantworten kann, haben Sie noch Gelegenheit zu einem ärztlichen Gespräch.

Bei der Untersuchung macht eine Röntgenassistentin von Ihren Brüsten je zwei Aufnahmen. Dabei wird Ihre Brust zwischen Platten gedrückt. Je flacher die Brust gedrückt wird, desto niedriger

ist die Strahlendosis und desto aussagekräftiger ist die Aufnahme. Das kann unangenehm oder auch schmerzhaft sein. Krebs kann dadurch nicht ausgelöst werden.

WAS GESCHIEHT NACH DER UNTERSUCHUNG?

Die Mammographie-Aufnahmen werden in den folgenden Tagen sorgfältig ausgewertet. – Zwei Ärztinnen oder Ärzte analysieren unabhängig voneinander die Aufnahmen Millimeter für Millimeter. Sie sollen dabei möglichst keine Veränderung übersehen, aber auch keinen harmlosen Schatten als verdächtigen Befund werten. Auffällige Befunde werden mit einem weiteren Spezialisten oder einer weiteren Spezialistin beraten. Allen Verantwortlichen ist bewusst, dass die Zeit des Wartens für Sie belastend sein kann. Der Brief mit dem Ergebnis sollte Ihnen innerhalb von sieben Werktagen nach der Untersuchung vorliegen. Manchmal kann es aber zu unvorhergesehenen Verzögerungen kommen. In den meisten Fällen wird die Aufnahme keinen verdächtigen Befund ergeben. Dann bekommen Sie in zwei Jahren die nächste Einladung zur Mammographie. Aber bitte denken Sie daran: Trotz aller Sorgfalt kann ein bösartiger Tumor in der Mammographie nicht sichtbar sein, oder er wächst erst in den zwei Jahren bis zur nächsten Untersuchung heran. Selten kann auch ein Tumor von den beiden Ärztinnen und Ärzten unabhängig voneinander übersehen werden. Sie sollten sich direkt an eine Ärztin oder einen Arzt wenden, wenn Ihnen in der Zeit bis zur nächsten Mammographie Veränderungen in Ihrer Brust auffallen, wie etwa:

→ tastbare Knoten, Dellen oder Verhärtungen der Haut,

→ sichtbare Verformungen, Hautveränderungen oder Einziehungen der Brustwarze,

→ Blutungen oder andere Absonderungen aus der Brustwarze.

WIE GEHT ES NACH EINEM VERDÄCHTIGEN BEFUND WEITER?

Wenn die Ärztinnen oder Ärzte einen verdächtigen oder unklaren Befund entdecken, werden Sie erneut eingeladen, damit der Befund abgeklärt werden kann. Dafür wird die Brust gezielt geröntgt oder mit Ultraschall untersucht. Lässt sich der Befund nicht eindeutig klären, wird die Entnahme einer Gewebeprobe empfohlen. Dabei wird unter lokaler Betäubung eine dünne Hohlnadel durch die Haut zu der auffälligen Stelle in der Brust gelegt. Durch diese Nadel werden dann mehrere kleine Gewebezylinder entnommen. Diese so genannte Stanzbiopsie ist ein kleiner und in den allermeisten Fällen komplikationsloser Eingriff. Das entnommene Gewebematerial wird anschließend von einer speziell geschulten Pathologin oder einem Pathologen unter dem Mikroskop begutachtet.

Früher glaubte man, dass sich die Krankheit noch verschlimmern würde, wenn durch die Nadel Tumorzellen gestreut werden. Dies hat sich ebenso wenig bestätigt wie die Befürchtung, dass der Tumor durch die zusätzliche Luftzufuhr einen Wachstumsschub bekommen könnte.

Insgesamt kann bei etwa fünf von sechs auffälligen Befunden Entwarnung gegeben werden. Dann gilt dasselbe wie nach einer unauffälligen Mammographie: Sie bekommen in zwei Jahren die nächste Einladung, sollten aber bis dahin Veränderungen ernst nehmen.

Falls sich der Verdacht auf Brustkrebs jedoch bestätigt, wird die Ärztin oder der Arzt der Screeningeinheit das weitere Vorgehen mit Ihnen besprechen. Selbstverständlich wird Sie auch Ihre Frauen- oder Hausärztin oder Ihr Frauen- oder Hausarzt hier weiterhin betreuen.

WELCHE VORTEILE UND NACHTEILE GIBT ES?

Eine Screening-Mammographie hat – wie jede medizinische Maßnahme – Vor- und Nachteile. Um möglichst deutliche Vorteile und möglichst geringe Nachteile zu haben, wurde das qualitätsgesicherte Mammographie-Screening-Programm eingeführt. Die Mehrzahl der Fachleute geht davon aus, dass dieses Programm für Frauen, die daran teilnehmen, mehr Vorteile als Nachteile bietet. Die Erfahrungen aus Ländern, die schon lange ein Screening-Programm anbieten, wie die Niederlande, Großbritannien und Schweden, bestätigen dies. Deshalb wurde auch in Deutschland so ein Programm eingeführt.

Ihre individuelle Bilanz aus Vor- und Nachteilen kann jedoch von der durchschnittlichen Bilanz abweichen. Der Grund: Für Frauen mit besonders vielen Risikofaktoren sind die Vorteile tendenziell deutlicher, für Frauen mit besonders wenigen Risikofaktoren tendenziell weniger deutlich.

Wichtig ist auch, wie Sie die Vor- und Nachteile ganz persönlich für sich bewerten, das heißt, ob Ihnen bestimmte Vor- oder Nachteile besonders am Herzen liegen.

Ein Nachteil ist,

- → wenn ein auffälliger Befund, der sich später als unbegründet herausstellt, beunruhigt, insbesondere wenn Gewebe entnommen wird, das sich nachträglich als gutartig herausstellt,
- → wenn ein bösartiger Tumor gefunden und behandelt wird, der nicht mehr heilbar ist und sich dadurch die Leidenszeit, aber nicht die Lebenszeit verlängert,
- → wenn ein Tumor gefunden und behandelt wird, der niemals Probleme bereitet hätte.

Ein Vorteil ist,

- → wenn durch das frühe Entdecken ein bösartiger Tumor schonender behandelt wird und zum Beispiel die Brust erhalten und auf eine Chemotherapie verzichtet werden kann,
- → wenn ein bösartiger Tumor in einem heilbaren Stadium gefunden wird, der ohne Untersuchung zum Tod geführt hätte.

WAS HABEN SIE KONKRET ZU ERWARTEN?

Folgende Zahlen, die auf Erfahrungen aus anderen Ländern und auf wissenschaftlichen Untersuchungen beruhen, sollen Ihnen eine konkrete Vorstellung davon geben, wie Vor-und Nachteile über das gesamte Programm in etwa statistisch verteilt sind:

- Von 200 Frauen, die 20 Jahre lang jedes 2. Jahr am Mammographie-Screening-Programm teilnehmen, erhalten 140 Frauen in 20 Jahren keinen verdächtigen Befund. 60 Frauen bekommen einen Befund, dem nachgegangen werden sollte.

- Von diesen 60 Frauen erhalten 40 bei der ergänzenden Untersuchung Entwarnung, 20 Frauen wird eine Gewebeentnahme empfohlen.

- Von diesen 20 Frauen stellt sich bei 10 Frauen der Verdacht als unbegründet heraus. 10 Frauen erhalten die Diagnose Brustkrebs im Screening, von den übrigen 190 Frauen erhalten 3 Frauen in den 20 Jahren zwischen zwei Screeningrunden ebenfalls die Diagnose Brustkrebs.

- Von diesen insgesamt 13 Frauen mit der Diagnose Brustkrebs sterben 3 Frauen an Brustkrebs, 10 Frauen sterben nicht an Brustkrebs.

- Von diesen 10 Frauen hätte 1 Frau ohne Mammographie zu Lebzeiten nichts von ihrem Brustkrebs erfahren, 8 Frauen wären auch ohne Teilnahme am Mammographie-Screening-Programm erfolgreich behandelt worden – ein Teil davon jedoch mit einer belastenderen Therapie. 1 von 200 Frauen wird dank ihrer regelmäßigen Teilnahme vor dem Tod durch Brustkrebs bewahrt.

WAS PASSIERT MIT IHREN DATEN?

Alle am Screening Beteiligten sorgen dafür, dass Ihre Daten mit der größtmöglichen Vertraulichkeit und Sicherheit behandelt werden. Unbefugte Einrichtungen und Personen haben keine Möglichkeit, Ihre Daten einzusehen. Für die wissenschaftliche Auswertung des Programms werden Ihre Daten unkenntlich gemacht. In der Krebsfrüherkennungs- Richtlinie des Gemeinsamen Bundesausschusses ist geregelt, wie die Daten erhoben, verarbeitet, genutzt und wie lange diese aufbewahrt werden. Im Übrigen gelten die nach Bundesdatenschutzgesetz und Sozialgesetzbuch festgelegten Rechte auf Auskunft (§§ 6, 19 und 34 BDSG bzw. § 83 SGB X) und auf Berichtigung, Löschung oder Sperrung (§§ 20 und 35 BDSG bzw. § 84 SGB X).

Diese Information in anderen Sprachen finden Sie unter:
www.mammo-programm.de
