# Supplementary material for: Effects of different information brochures on women’s decision-making regarding mammography screening: study protocol for a randomized controlled questionnaire study
Source: Trials. 2013 Oct 1;14:319. doi: 10.1186/1745-6215-14-319 (PMC3851440; doi:10.1186/1745-6215-14-319)
Supplement: Additional file 4 — Statement Ethikkommission.pdf (statement ethics committee of the university hospital, Heinrich-Heine University of Duesseldorf). [file 1745-6215-14-319-S4.pdf]

MAMMOGRAPHIE
SCREENING
PROGRAMM

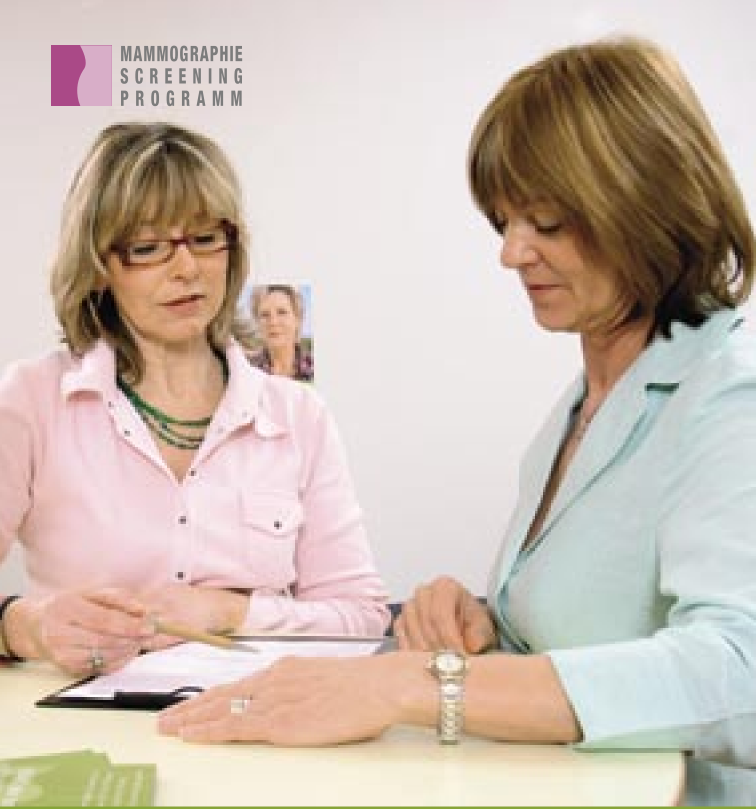

Informationen zum MAMMOGRAPHIE-SCREENING

Programm zur Früherkennung
von Brustkrebs für Frauen
zwischen 50 und 69 Jahren

Programm zur Früherkennung von Brustkrebs

Mit Ihrer persönlichen Einladung zur Teilnahme an dem Programm zur Früherkennung von Brustkrebs durch Mammographie-Screening erhalten Sie auch dieses Merkblatt. Sie werden über die Hintergründe, Ziele, Inhalte und Vorgehensweise dieses Programms informiert. Die Teilnahme an diesem Programm ist freiwillig.

Brustkrebs ist in Deutschland die häufigste Krebserkrankung bei Frauen. Etwa zehn Prozent aller Frauen erkranken im Laufe ihres Lebens daran, die meisten nach dem 50. Lebensjahr.

Daher wurde beschlossen, ein Programm zur Früherkennung von Brustkrebs durch Mammographie-Screening einzuführen. Ein solches Programm, das nach den EU-Leitlinien höchsten Qualitätsanforderungen entspricht, hat es in Deutschland bisher nicht gegeben. Zu den hohen Qualitätsanforderungen gehört beispielsweise, dass

- jede Röntgenaufnahme der Brust (Mammographie) von zwei besonders geschulten Ärzten beurteilt wird,
- jeder Arzt routinemäßig die Mammographien von mindestens 5.000 Frauen im Jahr beurteilt,
- im Falle einer Verdachtsdiagnose die erforderliche Abklärung innerhalb des Früherkennungsprogramms stattfindet. Die Ergebnisse werden von allen beteiligten Ärzten gemeinsam ausgewertet und das weitere Vorgehen besprochen,
- nur besonders qualifizierte Einrichtungen dieses Programm durchführen dürfen.

- Die Kosten werden im Rahmen Ihres Krankenversicherungsschutzes übernommen.
- Wenn Sie eine Krankenversicherungskarte haben, bringen Sie diese bitte zur Untersuchung mit.
- Sollten Sie an dem genannten Termin verhindert sein, wenden Sie sich bitte für eine Terminverschiebung an die einladende Stelle.
- Weitere Fragen können Ihnen gerne in der in Ihrer Einladung genannten Untersuchungsstelle (Mammographie-Einheit) beantwortet werden.

Was ist Mammographie-Screening?

Mit dem Begriff ‚Mammographie-Screening' wird ein Programm zur Früherkennung von Brustkrebs bezeichnet. Dadurch soll Brustkrebs möglichst frühzeitig bei Frauen entdeckt werden, bei denen noch keine Anzeichen für diese Erkrankung vorliegen.
In Abständen von zwei Jahren werden Röntgenaufnahmen der Brust (Mammographie) erstellt. Bei unauffälligem Befund werden Sie in zwei Jahren wieder eingeladen. Sollte sich aus den Röntgenaufnahmen der Verdacht auf eine Krebserkrankung ergeben, werden weitere Untersuchungen zur Abklärung dieses Verdachts vorgenommen. Dieses Programm hat sich zum Ziel gesetzt, die Zahl der Brustkrebstodesfälle in Deutschland zu senken und bei einer Krebserkrankung eine schonendere Behandlung zu ermöglichen. Daher werden strenge Qualitätsanforderungen an das Screening-Personal, die teilnehmenden Ärzte und die Geräte gestellt und die gegebenenfalls notwendigen Schritte zur Abklärung eines Brustkrebsverdachts aufeinander abgestimmt.

Welche Frauen können teilnehmen?

Es werden alle Frauen zwischen 50 und 69 Jahren im regelmäßigen Abstand von zwei Jahren auf der Grundlage von Angaben der Meldebehörden persönlich eingeladen.

Wenn Sie in den letzten zwölf Monaten bereits eine Mammographie erhalten haben, wenden Sie sich bitte an die einladende Stelle, um das weitere Vorgehen zu klären.

Sollten Sie zum Zeitpunkt der Einladung nachfolgende Beschwerden oder Symptome an Ihrer Brust haben, wenden Sie sich bitte direkt an Ihre behandelnde Ärztin oder Ihren behandelnden Arzt:

- tastbare Knoten,
- Dellen oder Verhärtungen der Haut,
- äußerlich sichtbare Verformungen,
- Hautveränderungen oder Einziehungen der Brustwarze,
- Blutungen oder andere flüssige Absonderungen aus der Brustwarze.

Sollten Sie aufgrund einer Brustkrebserkrankung in ärztlicher (Nach-)Behandlung sein, setzen Sie sich bitte mit der einladenden Stelle in Verbindung. Ein Anspruch auf Teilnahme am Programm besteht dann nicht, sodass Sie nicht mehr eingeladen werden.

Welche Vorteile bringt Ihnen die Teilnahme?

Durch die Mammographie können sehr kleine Krebsherde frühzeitig erkannt werden, bevor sie tastbar sind oder andere Symptome hervorrufen.
Die frühe Erkennung verbessert die Behandlungsmöglichkeiten und die Chancen einer Heilung.
Mit der regelmäßigen Teilnahme an diesem Programm erhöhen Sie daher Ihre Aussichten auf weniger eingreifende und belastende Behandlungsmethoden und auf eine dauerhafte Heilung.

Wie sicher ist die Mammographie?

Die Mammographie ist die derzeit beste Screeningmethode zur Früherkennung von Brustkrebs in der Altersgruppe der 50- bis 69-jährigen Frauen. Dennoch können mit der Mammographie nicht alle Brustkrebserkrankungen erkannt werden.
In seltenen Fällen kann auch im Zeitraum zwischen zwei Mammographien ein Brustkrebs entstehen und zu Beschwerden oder Symptomen führen.
Durch den Einsatz hochwertiger und regelmäßig überprüfter Röntgengeräte wird die Strahlenbelastung der Mammographie gering gehalten. Die durchschnittliche Strahlenbelastung einer Mammographieuntersuchung entspricht etwa zehn Prozent der durchschnittlichen natürlichen jährlichen Strahlenbelastung in Deutschland.
Der Nutzen der Untersuchung in dieser Altersgruppe unter den Voraussetzungen dieses qualitätsgesicherten Programms ist deutlich höher einzuschätzen als das Risiko, dass durch die Röntgenstrahlen bei der Mammographieerstellung Krebs ausgelöst werden kann.

Welche Grenzen und möglichen Nachteile bestehen bei der Mammographie?

In manchen Fällen ist die Erkrankung trotz frühzeitiger Erkennung und Behandlung nicht aufzuhalten.
Bei einem Verdacht auf eine Krebserkrankung kann durch die Abklärungsuntersuchungen eine Belastung entstehen (zum Beispiel durch Entnahme von Gewebeproben), auch wenn dieser Verdacht sich dann nicht bestätigt.
In einigen Fällen wird ein Brustkrebs entdeckt, der auch bei späterer Entdeckung noch erfolgreich behandelbar wäre.

Von jeder Brust werden zwei Röntgenaufnahmen angefertigt.

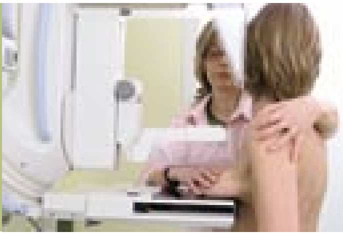

Was erwartet Sie bei der Mammographie?

Die Untersuchung dauert nur wenige Minuten. Jede Brust wird zwischen zwei Plexiglasplatten gelegt und kurz zusammengedrückt. Dies kann unangenehm, manchmal auch schmerzhaft sein. Dieser Druck ist notwendig, weil nur so gut beurteilbare Bilder mit der geringst möglichen Strahlenbelastung erzielt werden können.
Von jeder Brust werden zwei Röntgenaufnahmen angefertigt. Ihre Aufnahmen werden immer von mindestens zwei besonders erfahrenen und geschulten Ärztinnen/Ärzten unabhängig voneinander beurteilt.

Wie erhalten Sie das Ergebnis?

Das Untersuchungsergebnis wird Ihnen in der Regel innerhalb von sieben Werktagen nach Erstellung der Mammographie übermittelt.
Bei etwa 95 Prozent aller Frauen zeigt die Mammographie keinen Hinweis auf Brustkrebs. Diesen Frauen wird schriftlich mitgeteilt, dass sie in zwei Jahren wieder unaufgefordert eingeladen werden.
Auf gutartige Veränderungen, die nur äußerst selten ein Risiko darstellen, wird in der Mitteilung nicht hingewiesen

Die Mammographie ist auffällig – was passiert dann?

Zeigen sich in der Röntgenaufnahme Veränderungen, die auf eine Brustkrebserkrankung hinweisen können, sind weitere Untersuchungen notwendig. In diesem Fall erhalten Sie mit dem Ergebnis Ihrer Mammographie eine Einladung zur kurzfristigen weiteren Abklärung.
Die Ergebnisse der Abklärungsuntersuchungen werden von allen beteiligten Ärzten gemeinsam ausgewertet und besprochen. Bisherige Erfahrungen zeigen, dass sich durch die Untersuchungen bei circa 80 Prozent der Frauen, die wegen einer auffälligen Mammographie erneut einbestellt wurden, der Brustkrebsverdacht nicht bestätigt.

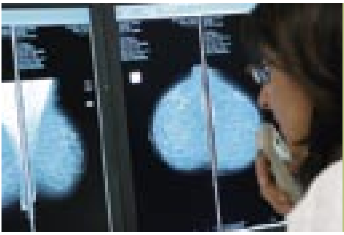

Bei etwa 95 Prozent aller Frauen zeigt die Mammographie keinen Hinweis auf Brustkrebs.

Was erwartet Sie bei der Abklärungsuntersuchung?

Ärztliches Gespräch

Als Erstes bespricht eine Ärztin oder ein Arzt Ihre Röntgenaufnahmen mit Ihnen und informiert Sie über die notwendigen weiteren Untersuchungen. Zu dem Gespräch können Sie nach Rücksprache mit der Ärztin/dem Arzt eine Person Ihres Vertrauens mitbringen.

Tastuntersuchung

Die Ärztin oder der Arzt tastet Ihre Brust sorgfältig ab.

Sonographie/Ultraschalluntersuchung

Bei dieser Untersuchung werden mittels Schallwellen Bilder des Brustgewebes erzeugt. Bei manchen Veränderungen kann die in der Mammographie entdeckte Auffälligkeit hierdurch ergänzend beurteilt werden.

Zusätzliche Röntgenaufnahmen

Bei einigen Veränderungen ist eine zusätzliche Röntgenaufnahme der Brust (zum Beispiel Vergrößerung) erforderlich. Damit lassen sich bestimmte auffällige Gewebebezirke genauer darstellen und beurteilen.

Ergebnis

Im Anschluss an diese Untersuchungen bespricht die Ärztin oder der Arzt das Ergebnis mit Ihnen. Bei den meisten Frauen kann ein Brustkrebsverdacht ausgeschlossen werden.

Biopsie

Eine Gewebeprobe (Biopsie) zur abschließenden Beurteilung eines auffälligen Befundes ist nur bei wenigen Frauen erforderlich.

Dazu wird nach örtlicher Betäubung mit einer Hohlnadel etwas Gewebe aus dem auffälligen Bezirk der Brust entnommen und von einem Spezialisten beurteilt. Das Ergebnis liegt nach wenigen Tagen vor und wird Ihnen in einem persönlichen Gespräch mitgeteilt. Die/der von Ihnen angegebene Ärztin/Arzt erhält ebenfalls das Ergebnis.

Das abschließende Ergebnis der Untersuchungen erhalten Sie in der Regel innerhalb von zwei Wochen nach der Einladung zur Abklärung in der mit Ihnen vereinbarten Weise. Sollte sich der Verdacht auf eine Krebserkrankung bestätigen, können Sie das weitere Vorgehen mit der Ärztin/dem Arzt, die/der die Abklärungsuntersuchungen durchgeführt hat, besprechen. Auch dazu können Sie nach Rücksprache mit der Ärztin/dem Arzt eine Person Ihres Vertrauens mitbringen.

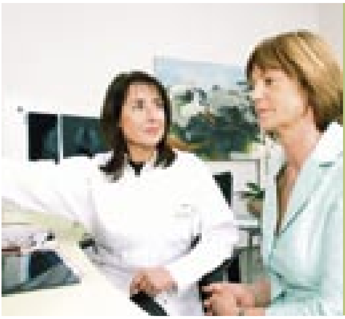

Als Erstes bespricht eine Ärztin oder ein Arzt Ihre Röntgenaufnahmen mit Ihnen und informiert Sie über die notwendigen weiteren Untersuchungen.

Wofür brauchen wir Ihre Daten, und wie sind Ihre Daten geschützt?

Dieses qualitativ hochwertige Früherkennungsprogramm wird allen Frauen zwischen 50 und 69 Jahren angeboten. Daher werden für die Einladung Daten aus den amtlichen Melderegistern (Namen und Anschrift, Geburtsdaten) verwendet. Die datenschutzrechtlichen Anforderungen werden dabei selbstverständlich eingehalten. Aus diesen Meldedaten werden zwei für Sie geltende, nicht wieder entschlüsselbare Kennnummern gebildet, die zur Organisation der Einladungen und zur Überprüfung der Qualität der Untersuchungen dienen.

Die amtlichen Meldedaten werden nach erfolgter Einladung gelöscht, unabhängig davon, ob Sie am Früherkennungsprogramm teilnehmen oder nicht. Die Maßnahmen zur Überprüfung der Qualität erfolgen in anonymisierter Form nur unter Verwendung der beiden Kennnummern. Wie bei allen sonstigen ärztlichen Behandlungen unterliegen Ihre Daten der ärztlichen Schweigepflicht.

In einer speziellen Maßnahme zur Überprüfung der Qualität der Untersuchungen wird festgestellt, ob bei Frauen in der Zeit zwischen zwei Screening-Mammographien Brustkrebs aufgetreten ist, der früher hätte entdeckt werden können. Zu diesem Zweck werden die Kennnummern der Teilnehmerinnen mit den Kennnummern in den zuständigen Krebsregistern regelmäßig abgeglichen. Das Krebsregister meldet die Kennnummern der Brustkrebsfälle (also nicht die Namen) an die einladende Stelle. Sollten Sie betroffen sein, übermittelt die einladende Stelle Ihre Kennnummer an die für Sie zuständige Screening-Einheit. Diese bittet Sie um Ihr Einverständnis, Ihre medizinischen Unterlagen, also insbesondere die Mammographieaufnahmen, an eine spezielle Einrichtung zur Qualitätsüberprüfung (Referenzzentrum) weiterleiten zu dürfen.

Worauf wir Sie noch hinweisen wollen

Sie sollten unbedingt auch während des zweijährigen Intervalls zwischen den Einladungen auf Veränderungen Ihrer Brust achten. Dazu gehören:

- tastbare Knoten,
- Dellen oder Verhärtungen der Haut,
- äußerlich sichtbare Verformungen,
- Hautveränderungen oder Einziehungen der Brustwarze,
- Blutungen oder andere flüssige Absonderungen aus der Brustwarze.

Wenn Sie solche Veränderungen bemerken, wenden Sie sich bitte unverzüglich an Ihre behandelnde Ärztin oder Ihren behandelnden Arzt.

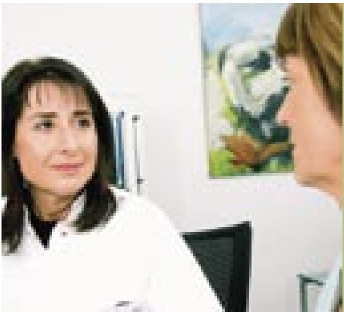

Wenn Sie solche Veränderungen bemerken, wenden Sie sich bitte unverzüglich an Ihre behandelnde Ärztin oder Ihren behandelnden Arzt.

Weitere Informationen erhalten Sie bei www.mammographie-screening.org oder bei der einladenden Stelle (Zentrale Stelle). Dort können Sie auch einen Termin vereinbaren.

Stand: 15.12.2003

Der Gemeinsame Bundesausschuss (G-BA) nach § 91 Abs. 5 SGB V beschließt die Krebsfrüherkennungs-Richtlinien.

Dieses Merkblatt ist Bestandteil der Krebsfrüherkennungs-Richtlinien und klärt über Hintergründe, Ziele, Inhalte und Vorgehensweise des Programms zur Früherkennung von Brustkrebs auf.

www.g-ba.de

Gemeinsamer Bundesausschuss
